# Cavity Enhanced Multi-Channels Gases Raman Spectrometer

**DOI:** 10.3390/s21113803

**Published:** 2021-05-31

**Authors:** Dewang Yang, Qingsheng Liu, Jinjia Guo, Lulu Wu, Andong Kong

**Affiliations:** 1 College of Information Science and Engineering, Ocean University of China, Qingdao 266061, China; yangdewang_lcu@126.com (D.Y.); liuqingsheng@stu.ouc.edu.cn (Q.L.); wululu1996@163.com (L.W.); kad669@163.com (A.K.); 2 Laser Institute, Qilu University of Technology (Shandong Academy of Sciences), Qingdao 266000, China

**Keywords:** cavity-enhanced Raman spectrometer, gas Raman spectra, multi-channels

## Abstract

Raman spectroscopy has the advantages of multi-component detection, with a simple device and wide concentration ranges, and it has been applied in environmental monitoring and gas logging. However, its low sensitivity has limited its further applications. In fact, the Raman signal is not weak, but the utilization efficiency of the Raman signal is low, and most of the signal is wasted. Given this, in this paper we report a cavity-enhanced multi-channel gas Raman spectrometer with an eight-sided cuvette. First, we simulated the Raman scattering intensity at angles from 30 degrees to 150 degrees. The simulation results showed that the signal intensity at an angle of 45° is 1.4 times that observed at 90°. Based on the simulation results, we designed a three-channel sample cell for higher sensitivity. The results of these experiments showed that the sensitivity could be increased by adding all signal together, and the limit of detection (LOD) for CO_2_ was 75 ppm, which is better than that of each channel. This paper thus presents a new method to enhance the Raman signal, which can be used in field applications.

## 1. Introduction

Raman spectroscopy has been widely used in environmental monitoring [1], gas logging [2] and other fields [3]. Although the traditional Raman system can only detect the components with high concentrations because of its low sensitivity, some Raman signal enhancement methods have been developed to improve the system’s sensitivity, such as surface-enhanced Raman spectroscopy (SERS), aiming to enlarge the scattering cross section [4,5]. However, this has rarely been used for the Raman scattering enhancement of gas detection [6]. The development of cavity-enhanced Raman spectroscopy (CERS) [7] aimed to increase the effective optical path or increase the laser power. CERS is widely applied in the enhancement of the Raman signal of gases.

Raman spectroscopy systems can be categorized as follows. (1) In the multiple reflection cavity [8] system, the laser oscillates in the multiple-reflection cavity and excites gases in the sample cell. The Raman signal intensity is increased with repeated excitations. The cavity types involved in this system include ellipsoidal cavities [9], right angle reflection cavities [10], near confocal cavities [11], concentric cavities [12] and near-concentric cavities [13]. (2) The laser resonator system combines a laser resonator with the sample cell to improve the Raman signal intensity [14,15]. (3) In the hollow fiber [16,17,18] system a hollow fiber is used as a sample cell, and the laser is coupled into the hollow fiber at the same time. The Raman signal generated in the fiber is transferred into the spectrometer for analysis. In summary, the hollow fiber method has the highest sensitivity due to its long effective distance and high collection efficiency. The multiple reflection cavity setup has a high level of robustness, although its sensitivity is lower than that of hollow fiber methods. The mean reason for this is its lower collection efficiency.

The detector of a Raman spectroscopy system includes either a charge-coupled device (CCD) [19] or a photomultiplier (PMT) [20]. The setup with a mounted CCD is able to detect multi-components simultaneously, and the detection cycle takes several minutes. The setup with a PMT exhibits a quick response (several seconds) and high sensitivity, although this system can only detect one component in one channel. It can be made to detect more components by adding channels. Most CERS systems use a mounted CCD to detect the Raman signal through one channel, and the radiation characteristics of the Raman scattering in this system are not yet clear.

In recent years, the use of CERS as a gas measurement tool has seen a rise in publications. In our preliminary work, we reported a limit of detection (LOD) of 52 ppm for CO_2_ based on a near-concentric cavity [13]. Li et al. have reported an LOD of 16 ppm for CO_2_ based on a near-confocal cavity [21]. Wang et al. achieved an LOD of 17.4 ppm for CO_2_ with the optical feedback frequency locking method [22]. Because of the various components (different detectors and lasers) and different acquisition parameters (integration times and number of accumulations), the LOD of each setup was different. Thus, it is quite meaningful to carry out the study of LOD enhancement based on CERS methods.

Therefore, with the aim of studying the radiation characteristics of Raman scattering in a CERS system and improving collection efficiency, we have simulated the scatting intensity at different angles. Furthermore, we invented a three-channel sample cell to enhance the collection efficiency. Based on this, the detection sensitivity will be further improved.

## 2. Principles and Experiments

### 2.1. The Simulation of the Near-Concentric Cavity

**Figure 1:**
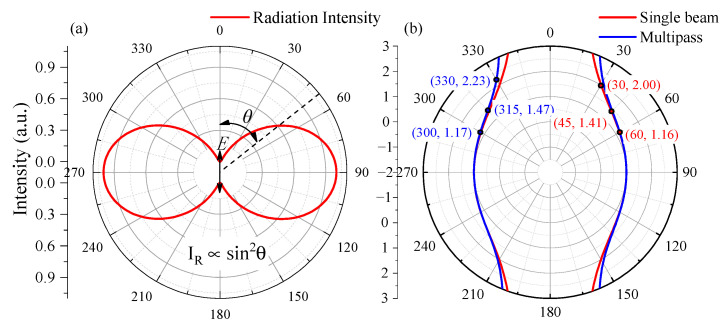
(**a**) Simulation result of angular distributions of the radiant intensity, *I_R_*; (**b**) simulation result of angular distributions of Raman scattering intensity with a single laser beam (red line) and multi-laser beam (blue line).

According to the classical theory, the most efficient source of electromagnetic radiation is an oscillating electric dipole. The radiation of an oscillating dipole is shown in Equation (1) [23]. The amplitude *E*_0_ of electric field intensity (*E*) of the radiation produced by the oscillating dipole at a distance r is given by

(1) $$xE_{0}=\frac{\pi \nu^{2}\mu_{0}\sin\theta }{\varepsilon_{0}r}\;E_{0}=\frac{\pi \nu^{2}\mu_{0}\sin\theta }{\varepsilon_{0}r}$$

where *υ* is the wavenumber of the radiation, *μ*_0_ is the magnitude of the oscillating dipole, *ε*_0_ is the permittivity of the medium and *θ* is the angle between the dipole and the direction of propagation. The radiant intensity, *I*, of the dipole within the given element of solid angle dΩ in a particular direction, defined by the angle *θ* (see Figure 1), is

(2) $$I(\theta )=\frac{d\Phi }{d\Omega }=\frac{\pi^{2}c\nu^{4}\mu_{0}^{2}\sin^{2}\theta }{2\varepsilon_{0}}$$

The distribution of the energy density has axial symmetry with the rotation axis running along the dipole. As can be seen in Figure 1a, the radiation intensity *I* is proportional to sin^2^*θ*. The radiation intensity parallel to the electric field is zero and the intensity perpendicular to the electric field direction is at the maximum. Therefore, in order to collect more Raman signal, we need to set the collection direction perpendicular to the polarization of laser.

**Figure 2:**
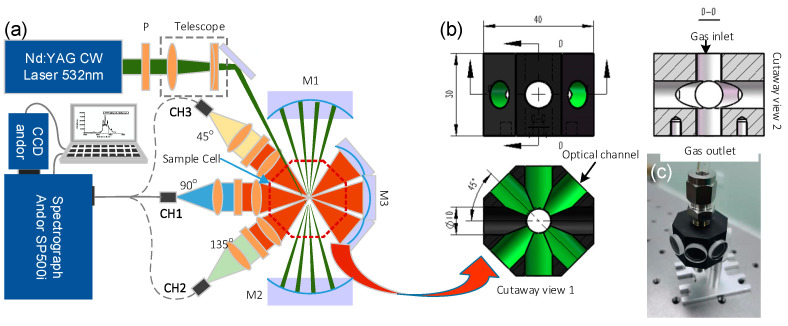
(**a**) Schematic of cavity-enhanced Raman spectrometer (CERS) prototype; (**b**) engineering drawing and cutaway view of the eight-sided cuvette; (**c**) image of the eight-sided cuvette.

The signal collection component (see in Figure 2a) is an f4 optical system, consisting of two identical achromatic lens with a focal length of 30 mm. The signal is coupled into a fiber bundle, consisting of 19 fibers with a 200-μm core diameter. The diameter of the effective aperture of the fiber bundle is about 1.5 mm. According to imaging principle of the f4 optical system, the effective length of 1.5 mm in the focus point could be collected into the optical fiber. When the angle between the collection direction and the optical axis direction is α, the effective length is equivalent to 1.5/sinα, as shown in Figure 1b. The signal intensity is weakest when the angle is 90° and the backward scattering signal intensity is strongest. The signal intensity at an angle of 30° is twice that at an angle of 90°, and the signal intensity at an angle of 45° is 1.4 times that at an angle of 90°.

As for the multiple reflection cavity, there are about 40 laser rays in the cavity, each of which has a different angle with respect to the collection direction. The relationship between the collection signal strength and the collection direction is represented by the blue line in Figure 1b. The signal intensity at 30 degrees is 2.23 times that of 90 degrees, and the signal intensity at angle of 45 degrees is 1.47 times that of 90 degrees. Therefore, the collection angle should be reduced when designing the collection light path.

### 2.2. Experimental Setup

Based on the simulated results, we adopted the near-concentric cavity to enhance Raman signals, as shown in Figure 2. The laser in this system was a diode-pumped solid-state laser (DPSS) with a wavelength of 532 nm and a power of 300 mW; the stability of the laser power was about 3%. The laser beam was reflected into a near-concentric cavity after beam shaping and polarization control. The telescope compressed the laser beam diameter to half (a planoconvex lens with a focal length of 100 mm and a planoconcave lens with a focal length of −50 mm). A half-wavelength plate (P) rotated the laser beam from P polarization to S polarization. The near concentric cavity was composed of two identical spherical mirrors with 25.4 mm diameter and 25 mm focal length. An eight-sided cuvette was mounted at the center of the cavity. The Raman signal was collected in three directions, and the signal collector was composed of two achromatic lenses with focal lengths of 30 mm and diameters of 25.4 mm. the spectrometer we used was an Andor SR500i (Abingdon, UK).equipped with 1200 g/mm grating. The Raman spectra were recorded using a CCD (Andor iVac 316, Abingdon, UK).

The eight-sided cell, with a size of 40 × 40 × 30 mm^3^, is shown in Figure 2b. The cell was made of aluminum alloy 6061; the surface of the cell was black-oxide-coated to decrease the stray light. An image of the cell is shown in Figure 2c. Two gas tubes, inlet and outlet, were mounted at the top and bottom of the chamber. Eight separate optical windows were mounted on the other eight sides of the chamber, respectively, for optical alignment; the optical windows were made of k9 glass with high anti-reflecting coating. The temperature was 25 degrees and the pressure in the cell was one bar.

The samples used in this study were purchased from Yantai Deyi Gas Co. Ltd., China. Their concentrations were measured by means of the gas chromatograph analysis method. The acquisition parameters of the CCD were as follows. The exposure time was 10 s, the accumulate cycle time was 10 s and the number of accumulates was 10 s. The Raman scattering signal was collected sequentially by channels 1, 2 and 3.

## 3. Results and Discussion

### 3.1. The Distribution of Signal Intensity with Scattering Angle

**Figure 3:**
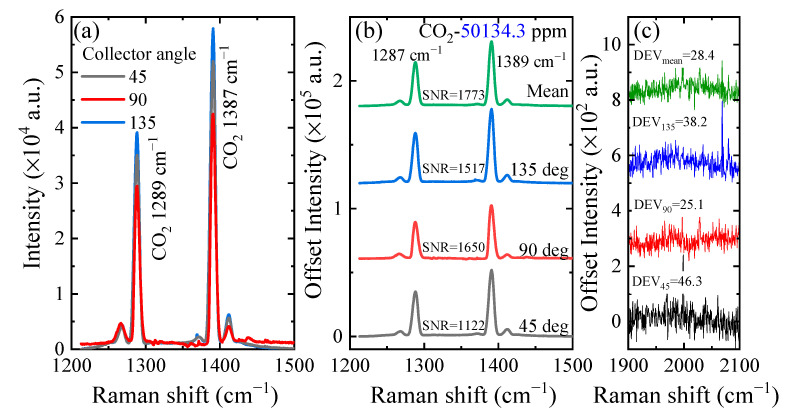
(**a**) The Raman spectra of CO_2_ at different collection angles. (**b**) Comparison of the signal-to-noise ratios (SNRs) of the four spectra; the three at the bottom are the spectra at different collection angles and the one at the top (in green) is the mean value of these three spectra. (**c**) The noise intensity of the four spectra.

According to the simulation results, we measured the intensity of gases’ Raman signals at different collection angles (shown in Figure 3). The sample was a mixed gas composed of CO_2_ and N_2_, and the concentration of CO_2_ was 50,134.3 ppm. The black line, red line and blue line in Figure 3a represent the Raman spectra of CO_2_ at the collection angles of 45 degrees, 90 degrees and 135 degrees, respectively. Two obvious Raman peaks of CO_2_ were located at 1289 cm^−1^ and 1387 cm^−1^. The Raman peak at 1387 cm^−1^ was stronger than the other one. Therefore, we use the stronger peak for quantitative analysis. Comparing the three spectra, the peak intensity of CO_2_ at 90 degrees was the smallest, and that at 135 degrees was strongest. In order to distinguish the differences in these spectra, the spectra were displayed at an offset of 60,000, as shown in Figure 3b. The green line in Figure 3b indicates the mean value of these three spectra. Moreover, the signal-to-noise ratio (SNR) was used to indicate the ratio of peak intensity to noise intensity, and the noise intensity of each spectrum is shown in Figure 3c. The signal intensity collected at 45 degrees was 1.42 times that collected at 90 degrees, which is almost equal to the theoretical value. The SNR of the mean spectrum was the largest (1773) among these four spectra, and the SNR of the spectrum at 90 degrees (1650) was larger than those at 45 degrees (1122) and 135 degrees (1517). Although the peak intensity of CO_2_ at 90 degrees was lower than the others, its noise intensity was the smallest among the three spectra.

### 3.2. The Spectra with Different Concentrations

**Figure 4:**
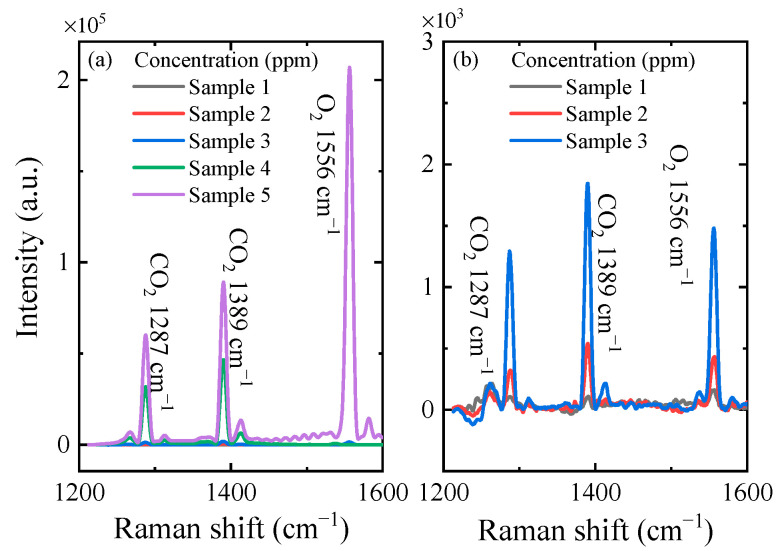
(**a**) The Raman spectra of sample 1 to sample 5. (**b**) The Raman spectra of sample 1 to sample 3.

Basis on this setup, we measured mixtures of gases with different concentrations of CO_2_ and O_2_ (shown in Figure 4). The components of the samples are shown in Table 1; the minimum concentration of CO_2_ was 98.8 ppm, and the maximum concentration of CO_2_ was 9.96%. The acquisition parameters of the CCD were as follows. The exposure time was 10 s, the accumulation cycle time was 10 s and the number of accumulates was 10. The peak intensities of CO_2_ and O_2_ became stronger as the concentrations increased. Figure 4b shows the spectra of CO_2_ and O_2_ at lower concentrations (100–2000 ppm). It is worth noting that the Raman peak of CO_2_ could be recognized at a low concentration of 98.8 ppm.

### 3.3. The Limit of Detection (LOD) of This System

**Figure 5:**
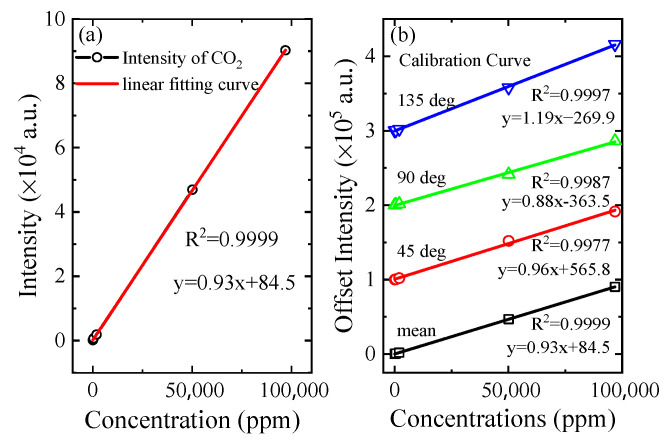
(**a**) The calibration curve of the mean spectrum. (**b**) The comparison of four calibration curves; three of them are the spectra at different collection angles and the bottom one (in black) is the mean values of these three spectra.

According to the relationship between the peak intensity of CO_2_ and its concentrations, the calibration curve is shown in Figure 5a. The hollow circles in that figure represent the peak intensity of CO_2_ at different concentrations, and the red line is a linear fitting curve. There is a good linear relationship between the peak intensities and the concentrations, and a correlation coefficient R^2^ = 0.9999. According to the 3σ standard, the standard deviation of the noise is 23.3, and the slope of the calibration curve is 0.93; the limit of detection (LOD) is 3σ/S = 75 ppm.

In addition, we created calibration curves based on the spectra collected at 45 degrees, 90 degrees and 135 degrees, respectively. These are shown in Figure 5b. In order to show the results more clearly and reduce the impact of overlapping areas, the four sets of data were shifted longitudinally and arranged in a picture. Comparing these four calibration curves, improvements in the following three aspects can be observed. (1) The linear correlation coefficient is improved, and the correlation coefficient of the calibration curve with three angles is up to 0.9997. After averaging the three curves, the correlation coefficient was increased to 0.9999. (2) The averaged data are closer to the point (0, 0), indicating that the averaged result is closer to the true value. (3) The detection limit is improved. According to the noise intensity of the three sets of spectra, the detection limits of the spectral data with the noise intensity of the collected signals at 45 degrees, 90 degrees and 135 degrees are 37.4, 40.7 and 37.0, respectively, and the detection limits are 116.9. ppm, 138.8 ppm and 93.3 ppm. Compared with the results of a previous report (16 ppm) [22], the LOD of this study was not prominent. This was mainly due to the different components of this setup and the different acquisition parameters. Even so, this study presents a further enhancement of the Raman scattering method on the basis of the CERS setup. The LOD could be further decreased to 80.4% of the original value (75.0 ppm/93.3 ppm) by using an eight-sided cell.

### 3.4. The Stability of This System

**Figure 6:**
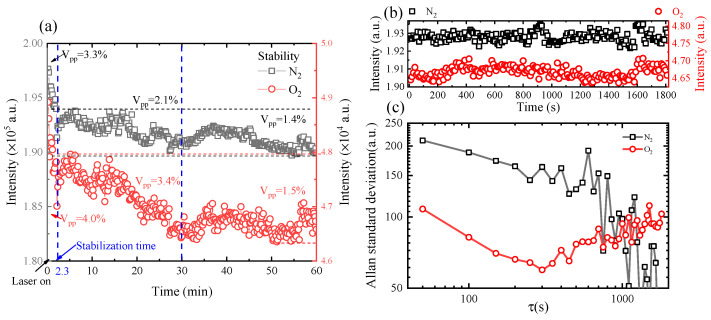
(**a**) The stability of this system over one hour of measurement; (**b**) the background correction data from 30 to 60 min; (**c**) the Allan standard deviation of N_2_ and O_2_ signals in the final 30 min.

In order to evaluate the stability of this system, we carried out a one-hour measurement using channel 2. The experimental conditions were as follows: the sample was air, the integration time was 10 s and the number of accumulations was one. The peak intensities of N_2_ and O_2_ are shown in Figure 6. The stability of this system was equal to (I_max_-I_min_)/I_avg_, in which I_max_, I_min_ and I_avg_ are the maximum, minimum and average values, respectively. The measurement time could be divided into three parts. The first part was the time before 2.3 min, during which the stability of O_2_ and N_2_ were 4% and 3.3%, respectively. The second part was the time from 2.3 min to 30 min, during which the stability of O_2_ and N_2_ were 3.4% and 2.1%, respectively. The third part lasted from 30 min to 60 min, during which the stability of O_2_ and N_2_ were 1.5% and 1.4%, respectively. This stability was mainly related to the stability of the laser power (about 3%). Therefore, the final stability of the system could reach 1.5% after half an hour of warming up. In this case, we extracted the signals from 30 to 60 min to calculate the Allan standard deviation. Before that, the data from 30 to 60 min were removed the background, and the results of this process are shown in Figure 6b. Based on this, the Allan standard deviation was calculated, as shown in Figure 6c.

## 4. Conclusions

Raman spectroscopy systems have been used as gas sensors in environmental monitoring, gas logging and gas detection. However, the sensitivity of commercial Raman systems is too low, and needs to be improved for further applications. In fact, the Raman signal is not weak, but the utilization efficiency of the Raman signal is low, and most of the signal is wasted. Given these facts, in this study we have developed and analyzed a multi-channel Raman system with an eight-sided cuvette. First, we simulated the relationship between the intensity of the Raman scattering signal and the scattering direction. The simulation results showed that the signal intensity at 30 degrees was 2.23 times that at 90 degrees, and the signal intensity at an angle of 45 degrees was 1.47 times that at an angle of 90 degrees. On this basis, a set of eight-sided sample cells was designed and processed. Two windows were used as laser transmission windows, and the other six windows served as signal collection windows to increase the efficiency of the signal collection. The experimental results showed that the intensity of the Raman signals collected in the 45-degree or 135-degree directions were about 1.4 times that of the lateral collection signal. After averaging the signals collected in the three directions, the resultant Raman signal exhibited the highest signal-to-noise ratio. The eight-sided sample cell can thus attain a signal intensity that is sufficient to achieve a limit of carbon dioxide detection as low as 75 ppm.

## Figures and Tables

**Table 1:** The components of samples.

Components	CO_2_	O_2_	N_2_
Sample 1	98.8 ppm	102.0 ppm	other
Sample 2	511.0 ppm	508.2 ppm	other
Sample 3	2028.7 ppm	1996.0 ppm	other
Sample 4	50,134.3 ppm	0	other
Sample 5	9.96%	28.00%	other

## Data Availability

Data sharing not applicable.

## References

[1] Tang H., Zhu C., Meng G., Wu N. (2018). Surface-enhanced Raman scattering sensors for food safety and environmental monitoring. J. Electrochem. Soc..

[2] Han X., Huang Z., Chen X., Li Q., Xu K., Chen D. (2017). On-line multi-component analysis of gases for mud logging industry using data driven Raman spectroscopy. Fuel.

[3] Kiefer J., Seeger T., Steuer S., Schorsch S., Weikl M.C., Leipertz A. (2008). Design and characterization of a Raman-scattering-based sensor system for temporally resolved gas analysis and its application in a gas turbine power plant. Meas. Sci. Technol..

[4] Sun M., Li B., Liu X., Chen J., Mu T., Zhu L., Guo J., Ma X. (2019). Performance enhancement of paper-based SERS chips by shell-isolated nanoparticle-enhanced Raman spectroscopy. J. Mater. Sci. Technol..

[5] Zeng F., Duan W., Zhu B., Mu T., Zhu L., Guo J., Ma X. (2019). Paper-based Versatile SERS Chip with Smartphone-based Raman Analyzer for Point of Care Application. Anal. Chem..

[6] Lascola R., Mcwhorter S., Murph S.H. Apparatus for SERS Analysis of Gases. 2015-04-28. https://www.researchgate.net/publication/281434903_Apparatus_for_SERS_Analysis_of_Gases.

[7] Hippler M. (2015). Cavity-enhanced Raman spectroscopy of natural gas with optical feedback cw-diode lasers. Anal. Chem..

[8] Petrov D.V. (2016). Multipass optical system for a Raman gas spectrometer. Appl. Opt..

[9] Hill R.A., Hartley D.L. (1974). Focused, Multiple-pass cell for Raman scattering. Appl. Opt..

[10] Hill R.A., Mulac A.J., Hackett C.E. (1977). Retroreflecting multipass cell for Raman scattering. Appl. Opt..

[11] Li X., Xia Y., Li Z., Huang J. (2008). Near-confocal cavity-enhanced Raman spectroscopy for multitrace-gas detection. Opt. Lett..

[12] Taylor D.J., Glugla M., Penzhorn R.D. (2001). Enhanced Raman sensitivity using an actively stabilized external resonator. Rev. Sci. Instrum..

[13] Yang D., Guo J., Liu Q., Luo Z., Yan J., Zheng R. (2016). Highly sensitive Raman system for dissolved gas analysis in water. Appl. Opt..

[14] Miles S.D. (1992). Gas Analysis System Having Buffer Gas Inputs to Protect Associated Optical Elements. U.S. Patent.

[15] Beck K.F., Owen C.V. (1998). Raman Gas Analysis System with Cavity/Boss Assembly for Precision Optical Alignment. U.S. Patent.

[16] Markin A.V., Markina N.E., Goryacheva I.Y. (2017). Raman spectroscopy based analysis inside photonic-crystal fibers. Trac-Trend Anal. Chem..

[17] Hanf S., Keiner R., Yan D., Popp J., Frosch T. (2014). Fiber-enhanced Raman multigas spectroscopy: A versatile tool for environmental gas sensing and breath analysis. Anal. Chem..

[18] Hanf S., Bögözi T., Keiner R., Frosch T., Popp J. (2015). Fast and highly sensitive fiber-enhanced Raman spectroscopic monitoring of molecular H2 and CH4 for point-of-care diagnosis of malabsorption disorders in exhaled human breath. Anal. Chem..

[19] Wang P., Chen W., Wan F., Wang J., Hu J. (2020). A review of cavity-enhanced Raman spectroscopy as a gas sensing method. Appl. Spectrosc. Rev..

[20] Buric M.P., Chen K.P., Falk J., Woodruff S.D. (2009). Improved sensitivity gas detection by spontaneous Raman scattering. Appl. Opt..

[21] Li X., Xia Y., Huang J., Zhan L. (2008). Diagnosis of multiple gases separated from transformer oil using cavity-enhanced Raman spectroscopy. Chin. Phys. Lett..

[22] Wang P., Chen W., Wan F., Wang J., Hu J. (2019). Cavity-enhanced Raman spectroscopy with optical feedback frequency locking for gas sensing. Opt. Express.

[23] Keresztury G., Chalmers J.M., Griffiths P.R. (2002). Raman Spectroscopy: Theory. Handbook of Vibrational Spectroscopy.

